# Bioorganometallic Compounds as Novel Drug Targets against Schistosomiasis in Sub-Saharan Africa: An alternative to Praziquantel?

**DOI:** 10.34172/apb.2022.029

**Published:** 2021-05-30

**Authors:** Cuma Cumisa Ndamse, Priscilla Masamba, Abidemi Paul Kappo

**Affiliations:** Molecular Biophysics and Structural Biology Group, Department of Biochemistry, University of Johannesburg, Kingsway Campus, Auckland Park 2006, South Africa.

**Keywords:** Bioorganometallic compounds, Praziquantel, Reactive oxygen species, Schistosoma mansoni, Schistosomiasis, Redox biology

## Abstract

Human schistosomiasis is a disease that mostly plagues the destitute of various tropical and sub-tropical countries, particularly in sub-Saharan Africa (SSA) and South America. It has significant effects on various health and economic-related matters. Globally, the burden of schistosomiasis has been controlled with a single chemotherapeutic drug, praziquantel (PZQ), which has recently demonstrated several clinical issues, including its inability to destroy juvenile schistosome worms and drug resistance because of its extensive use. The use of organometallic moieties in biological and medicinal chemistry has developed greatly and has led to their use in various anti-cancer and anti-infectious agents. The abundance of a range of organometallic compounds that can cause damage to the parasite has received tremendous feedback, with many already at clinical trials. The distinct redox biology of the schistosome parasite is a vulnerable element to the survival of the worm and has steered attempts toward the use of redox-directed bioorganometallic compounds. Disruption of the schistosome redox homeostasis through organometallic ions provides a novel drug target that could be used in overcoming the drawbacks of the mainstream drug and one that could possibly bypass the emergence of drug resistance.

## Introduction

Schistosomiasis is a parasitic disease resulting from blood-dwelling trematode worms of the genus *Schistosoma.*^1,2^ The main causative agents of the disease in humans are associated with five species: *Schistosoma mansoni, Schistosoma japonicum* and* Schistosoma haematobium*, which are responsible for over 90% of all *Schistosoma* infections,^3^ as well as the two lesser and more rare *Schistosoma mekongi*and *Schistosoma intercalatum.*^1,4,5^ *S. haematobium* is the cause of urogenital schistosomiasis, while *S. mansoni* and *S. japonicum* are associated with gastrointestinal and hepatosplenic schistosomiasis respectively.^1,6^ According to the World Health Organization (WHO), this water-borne disease is the second most prevalent neglected tropical disease (NTD) and is responsible for infection in over 1 billion people worldwide, with over 20 million people exhibiting chronic and severe forms of the disease, while others are free from any signs of the infection, with millions more that remain susceptible to exposure.^7-10^ The burden of the disease is evident in the mortality rates that tally over 200 000 deaths annually and the loss of over 10 million disability-adjusted life-years (DALYs).^5,11,12^ People residing in rural and disadvantaged urban areas in third world countries, where healthcare systems are underdeveloped, are most affected.

Although numerous programs have been directed at fighting the global prevalence of schistosomiasis, infection rates are still extremely high, especially among inhabitants of the sub-Saharan Africa (SSA) region.^6,7,12-15^ Despite constituting only 13% of the world’s population,^15^ SSA, which has the world’s greatest concentration of poverty, accounts for almost 93% (192 million) of all people infected with schistosomiasis worldwide.^12,13^ Approximately 77% of the estimated DALYs attributed to the disease annually are lost in this region alone.^6^ In 2003, Van der Werf stated that 37 and 38 SSA countries showed endemicity to *S. haematobium* and *S. mansoni* respectively, with the former showing an estimated infection prevalence of 26% while the latter had a prevalence of 18%.^16^ The top five countries grappling with schistosomiasis in SSA are Nigeria (25.83 million), the United Republic of Tanzania (15.24 million), the Democratic Republic of Congo (13.84 million), Ghana (12.4 million) and lastly, Mozambique (11.3 million).^7^ Over a decade after the implementation of various control and elimination programs, these countries still record the highest prevalence of the disease, with significant increases in the number of infected persons.^12,13^ Schistosome hotspots, where the prevalence and intensities of infection remain high despite repeated rounds of chemotherapy, have been identified in many countries, including Mali, Kenya, Tanzania and Cameroon. Many of these localities are near contaminated lakes and dams, where individuals live a pastoral lifestyle and frequently take part in activities such as bathing, recreational swimming and fishing. These results in very high re-transmission rates that cause prevalence in those areas to return quickly to the initial levels immediately after patients have undergone treatment.^8,13,17^

Women in their productive years and children living in endemic regions are most vulnerable to infection owing to their constant exposure to open and contaminated water bodies while performing daily chores such as washing laundry and dishes.^10^ In addition, persons heavily involved in agricultural, fishing and irrigation activities or living a pastoral lifestyle are highly exposed and at risk of contracting this infection. Different studies have shown the link between infected persons and several deleterious consequences on issues such as workers’ productivity, pregnancy outcome, cognitive development and growth.^1,5,6,18^ A 2017 Kenyan study by Kuta (unpublished) showed that *S. haematobium* infections in school-aged children resulted in anemia and stunted physical growth, which affect intellectual capacity and school attendance. Such issues not only affect the economies of countries, but also perpetuate a stringent cycle of poverty that hinders the livelihoods of those affected.^18^

## Parasite lifecycle and associated co-morbidities

Schistosomes have a complex lifecycle that introduces them to hostile and unfavorable conditions as they transition between intra-mammalian and intra-molluscan stages.^5^ A feature that distinguishes schistosomes from other trematodes is their dioeciousness, where the body of the male safely and permanently lodges the longer and thinner female within a gynaecophoric canal formed. In this permanent embrace, the schistosomes reside within the peri-vesicle (*S. haematobium*) or mesenteric (other species) venous plexus of the human host, where they survive through anaerobic glycolysis and are able to mate and produce hundreds to thousands of fertilized eggs per day. The ova have ciliated larvae that can produce proteolytic enzymes that allow the eggs to migrate to the lumen either of the bladder (*S. haematobium*) or of intestine (*S. mansoni* and *S. japonicum*). These eggs are either retained in the host’s tissues or shed into the environment through urine or feces and then deposited into water, where the eggs can stay viable for up to seven days. Once in water, the eggs hatch and release free-swimming miracidia that follow phototrophic and chemical cues to search for and find the intermediate molluscan host.^5^ Snail vectors differ for each of the Schistosoma species; *Biomphalaria glabrata*for *S. mansoni*, *Oncomelaniahupensis* for *S. japonicum* and *Bulinus truncates*for *S. haematobium.*^3,8,19^ This feature distinguishes and limits the geographical distribution of each *Schistosoma* species.^1^ Once each snail host has been penetrated, the miricadia shed the cilia plate to become primary sporocysts that multiply asexually into multicellular sporocysts, which later transform into cercarial larvae with a characteristic bifurcated tail. After four to six weeks of infection, the cercariae leave the snail through light stimuli and live in water for up to 72 hours in pursuit of a suitable definitive host. A single snail infected by one miricadia can shed thousands of cercariae every day over a period of months. Following a thermal and light gradient, the cercariae swim upward and are directed by chemical cues towards their human host.^1,5,11,20^ After locating a host, the cercariae remain on the skin for three to four days before releasing proteases that enable them to burrow into mammalian skin. Upon entry, they lose their tails and transform into schistosomulae with a double-lipid barrier that protects them against host immune responses.^2^ The young worms incorporate host proteins and various major histocompatibility complexes to help them migrate through the host’s circulation, which carries the cercariae to various organs, including the heart, lungs and liver.^6,10,21^ The schistosomula mature and develop into mature schistosomes, which are white or greyish cylindrical worms with a length of about 7-20 mm. These trematode worms have conspicuous suckers, a blind digestive tract as well as a complicated reproductive system and tegument. Within the peri-vesical venous or mesenteric plexus, these worms feed anaerobically on blood and globulins, while the resultant waste is regurgitated back into the host blood.^2,4,6,13,21^ Over a period of four to six weeks within the portal vein, the adult worms pair to form embraced couples that mate and lay eggs in their respective peri-vesicular or mesenteric destinations. Some African and oriental species can produce hundreds to thousands of ova daily. It is the lodging of these eggs, which instigates an inflammatory granulomatous response that results in disease pathology and scarring in the host before they are expelled through either urine or feces, completing the cycle.^1,6,13^ Some eggs can remain trapped within the host and can survive for three to five years and in some cases for over 30 years, resulting in severe and life-threatening pathology, which is primarily dependent on infection intensity and innate patient immune responses.^1,6^ Clinically, this may be manifest in three stages. An early stage is followed by a secondary or acute stage, known as Katayama fever, where mild symptoms that include fever, fatigue, myalgia, malaise, a non-productive cough and eosinophilia occur. Abdominal discomfort and cramps may subsequently appear because of the migration and positioning of the mature worms. Lastly, a more persistent and worrisome stage of the disease may lead to a chronic phase characterized by diarrhea, toxemia, hepatosplenomegaly, weight loss, dyspnea, portal hypertension and liver fibrosis.^3,6,21^ Genital schistosomiasis among women raises the risk of contracting HIV owing to scarring and inflammation on the genital epithelial tissue that leaves a breach in the physical barrier during sexual intercourse. Furthermore, CD4+ cells are reported to increase their concentration of HIV co-receptors during active schistosomiasis.^1^

Most alarming is the possible link between schistosomiasis and cancer. Lodging of the eggs in the venous plexus has been linked to the onset of bladder cancer, as the eggs result in inflammation that produces oxygen-derived free radicals.^22,23^ In the case of Schistosoma colitis, *S. japonicum* has been associated with the development of adenocarcinomas of the colon, while there is evidence supporting growing cases of colorectal cancer in areas where *S. mansoni-*related schistosomiasis is endemic and populations exhibit chronic illness.^24-26^ Lastly, *S. haematobium* has been labeled a Group 1 carcinogen, as it has been associated with the production of polycyclic hydrocarbons and amines, predisposing sufferers to bladder cancer.^27^

## The redox biology of schistosomiasis

Reactive oxygen species (ROS), which primarily include hydroxyl radical (OH^∙^), singlet oxygen (^1^O_2_), superoxide anion (O_2_-) and hydrogen peroxide (H_2_O_2_), are oxygen-free radicals that are associated with dualistic roles in biological systems, as they could either be harmful or beneficial to the living organism.^28,29^ ROS are generated during irradiation by X-ray, ultraviolet light and gamma rays, as products of metal-catalyzed reactions and pollutants in the atmosphere, during inflammation and as by-products of mitochondria-catalyzed electron transport transfer and other mechanisms. They are pertinent to various functions in the signaling of cellular systems, elimination of oxidative stress involving mitigating damage of lipids, nucleic acids and proteins and even in defenses against infectious agents.^21,29^ The harmful effects of these molecular fragments are meticulously controlled in each organism by balancing out their effects through enzymatic and non-enzymatic antioxidant action. The enzymatic antioxidants include superoxide dismutase (SOD), catalase and members of the glutathione and thioredoxin (Trx) families, while non-enzymatic antioxidants include small, redox-active molecules such as ascorbic acid, uric acid and vitamin E.^21,29^

During these reactions, superoxide anion, generated by metabolic processes or “oxygen activation” during physiological processes or irradiation, is considered a primary ROS that can interact with other molecules in enzyme- or metal-catalyzed reactions to form secondary ROS. The superoxide anion can form hydrogen peroxide through catalysis with SOD. The generation of free radicals is linked to redox-active metals such as copper and iron, which are largely used by the cell. The release of iron, which is normally maintained in the cell, is triggered by excess superoxide anions from “iron-containing” molecules and the released free iron [Fe(II)] is capable of participating in the Fenton reaction with the available hydrogen peroxide to generate highly reactive hydroxyl radicals capable of destroying macromolecules such as proteins, DNA and lipids. The net reaction describing the production of these radicals is the Haber-Weiss reaction. As indicated, superoxide anion is dismutated by SOD to H_2_O_2_, which is removable by catalase, glutathione peroxidase (GPx) and peroxiredoxin. Reducing equivalents of these structures are glutathione (GSH) and thioredoxin, respectively.^28,30,31^

To survive in the aerobic environment of the human host, schistosome worms not only have to withstand attack by ROS generated by the host’s cytotoxic antibody-dependent cell-mediated white cells; they are also vulnerable to the same molecules manufactured by their own systems through various mechanisms.^21,28,29,32^ In schistosome worms, hemoglobin digestion, which is an essential source of nutrients, is the main catabolic process that produces the indigestible and toxic heme (iron III protoporphyrin IX (Fe^III^ PPIX)). This is followed by concurrent influxes of ROS that induce oxygen-derived radical reactions and over-oxidation of macromolecules.^28,33-35^ These worms, like malarial parasites, evade this toxic heme through two detoxification pathways, biocrystallization of the heme into hemozoin and a thiol network.^33,34^ Studies have shown that eukaryotic organisms possess two major detoxifying systems meant to protect them from ROS. The GSH and Trx systems, composed of GSH reductase (GR) and GSH, as well as Trx reductase (TrxR) and Trx, which both have NADPH-dependent reducing equivalents, are thiol-dependent redox balance systems linked to the antioxidant network as well as cell growth and differentiation, synthesis of DNA and transcription factor growth regulation.^28,36-40^

The antioxidant capacity of schistosomes is more tightly regulated than that of its mammalian host because of the worm’s lack of catalase and the low activity of schistosome GPx proteins towards H_2_O_2,_ despite an abundance of SOD.^35,38,39,41^ This makes the parasite highly sensitive to hydrogen peroxide compared to the human host, which could explain the observation of increases in the hydrogen peroxide detoxification capacity and ability to resist ROS from multiple sources along the development of *S. mansoni* parasites within its human host, indicating an improvement in the antioxidant defenses of the worm. This also suggests that adult schistosomes are susceptible to more redox challenges than juvenile worms.^21,28^

Developmental regulation of schistosome redox capacity is supported by the low expression of antioxidant mRNAs such as GST, CT-SOD and GPx in schistosomula. This makes larval parasites more susceptible to lung- and skin-generated radicals activated by the immune cells than adult worms. This is indicative of an adaptive mechanism employed by these worms to avoid redox imbalance and parasite cell death induced by the host immune system. This complex and unique homeostasis mechanism in the schistosome redox pathway presents weak points in their biology that can be used as targets for drug development.

Since schistosomes, similar to other flatworms, lack TrxR and GR, all their functions are instead contained in a multifunctional enzyme called Trx glutathione reductase (TGR) that is responsible for reducing glutathione disulfide (GSSG) and Trx.^28,36^ Although this enzyme is also present in mammalian cells, it is mainly associated with reproduction and is found primarily in the testes, where it is associated with facilitating sperm maturation and plays a far lesser role in redox protection in other cells.^28,39^ Cloning of the enzyme from *S. mansoni* revealed that it is an essential selenoprotein, containing the highly reactive selenocysteine amino acid and unlike mammalian TrxR*, S. mansoni* TGR is incapable of reducing dehydroascorbic acid or ubiquinone and can instead catalyze deglutathionylation reactions. This is one of the selenoenzyme’s vital roles under oxidative stress, since the resulting increase in GSSG probes the S-glutathionylation of cysteine residues. The protection of redox-sensitive cysteine from irreversible over-oxidation to sulfinic or sulfonic acids helps to prevent overall loss of protein activity. Therefore, the deglutathionylation reactions are present as regulatory mechanisms in the cells of the worm to restore glutathionylated proteins to their normal activity after cellular redox homeostasis is achieved.^28^

Another vital redox enzyme in schistosomes is the methionine sulfoxide reductases (Msrs). Since methionine residues are at risk of being oxidized to methionine sulfoxide by H_2_O_2_ during oxidative stress, the formation of these proteins impairs the structural integrity and overall activity of these proteins.^28,37^ Such Msr proteins are not only considered important in the management of oxidative stress and the repair of damaged proteins, but are thought to be vital ROS scavengers. Met-O residues exist in diastereomeric forms, as *S*- and *R*-epimers (Met-*S*-O and Met-*R*-O), and these proteins are normally classified into two types according to their substrate stereospecificity. MrsA and MrsB proteins are functionally different; the former are linked to reducing free and peptide Met-S-O while the latter is associated with reducing peptide Met-R-O but is found to have low/weak activity on free Met-R-O. In addition, MsrA has the ability to reduce other sulfoxide compounds such as sulindac and dimethyl sulfoxide.^28,42^ While two types of MsrB proteins exist in humans as selenocysteine and Cys-containing MrsB forms*, S. mansoni* worms are reported to have two genes similar to MsrB genes (MsrB1 and Msr2), both of which are cysteine-containing. Despite this, observations of the active sites of both proteins show that MsrB1 contains two cysteine residues in contrast to the one cysteine residue in MsrB2.^28^ Due to this, catalytic mechanisms of MsrB1 and MsrB2 of *S. mansoni* have been proposed. The catalytic cysteine residues in the Msr proteins interact with Met-O and can generate a sulfenic acid intermediate on the residue to release a free or peptide-bound protein. The second cysteine residue is linked to recycling and attacking the sulfenic acid and converting it into intramolecular disulfide that can be reduced by Trx. In MsrB2, the recycling cysteine is replaced by a threonine residue, thus requiring direct reduction of the sulfenic acid intermediate by Trx,^28^ although it has been suggested that other cysteine residues located in other positions of the Msr protein could act as a cys-recycling residue. Both MsrB proteins in *S. mansoni* maintain separate substrate specificities, with MsrB1 having a higher affinity for free Met-S-SO and MsrB2 preferring peptide Met-S-SO.

MsrB proteins are also classified according to the presence or absence of zinc residues, with both MsrB1 and MsrB predicted to contain essential and conserved zinc binding cys-motifs that are significant in the maintenance of the 3-D structure. Mutation of the residues within this motif can result in the absolute loss of metal-binding and catalytic activity.^28,37^ Expression of both MsrB1 and MsrB2 in schistosomiasis most likely occurs during the developmental stages of the worm, where the most prominent expression of both proteins is seen in eggs and the lowest expression is assumed to be in schistosomula. The increase in Msr protein synthesis in eggs is assumed to assist in the neutralization of ROS produced in the granuloma because of host immune mechanisms. Similar orthologs of MsrA are yet to be reported in the *S. mansoni* genomic sequence. Although no known drug targets are aimed at *S. mansoni*Msr proteins,^28^ the distinguished mechanisms between its hosts and its possible significance in the worm redox homeostasis mechanisms may provide a niche for redox-based anti-schistosome drugs.

Over the years, different chemotherapeutic agents have been mass-distributed to areas where schistosomiasis is endemic as part of integrated efforts to control and eventually eliminate the burden posed by the disease.^17^ In 2015, the WHO estimated that approximately 218 700 000 people required treatment for schistosomiasis, of which over 90% lived in SSA.^17,43^ Due to initial arguments that special schistosomiasis control strategies were not a priority, the availability of anti-schistosome drugs was limited in various health facilities, especially in this region.^7^ Several studies have contradicted this argument,^13,15,43,44^ some going as far as to say that not only are many of these countries far from controlling morbidity and mortality rates, but the current disease burden makes total eradication impossible.^45^ Despite the success of schistosomiasis control programs in countries such as Egypt, China and some SSA countries,^3,5,13^ similar initiatives have proven futile in other countries such as Madagascar, Senegal and Cameroon,^45^ as they cannot achieve control without foreign donor funding.^13^

In the 1980s and early 1970s the primary focus was on transmission control programs that relied on molluscicides, which proved not only to be labor-intensive but also entailed the extensive use of expensive chemicals, and required environmental control and management, until the WHO Expert Committee introduced a chemotherapeutic solution that would be accessible to various endemic countries.^5,7,46^ Early anti-schistosomal drugs such as antimony salts had several severe to lethal effects that required extensive attention to patients’ health.^6^ The introduction of safe, effective and simple drugs, such as the heavily relied-upon praziquantel (PZQ) that could be used for large-scale treatment for at-risk population groups in conjunction with improved sanitation, hygiene education and access to safe water, introduced a control strategy targeted at reducing the disease burden through large-scale preventive chemotherapy.^6,8,43,44,47^ However, this intervention method has been shown to be limited, as infection rates continue to rise in endemic regions and evidence of drug resistance raises concerns both clinically and scientifically. The introduction of metal-based compounds in chemical biology has opened the door to developing newer and novel drug treatment options against the disease.

## Organometallic and bioorganometallic chemistry

In the past four decades, there has been significant progress in the development of organometallic compounds for biological and medicinal purposes since their extensive use in industrial processes such as catalysis.^48-50^ Despite the presence of naturally occurring organometallic compounds such as hydrogenases found in reversible proton-electron conversions in biological systems as well as cobalamins (vitamin B_12_) and its derivatives, the application of metal ions and metal complexes in medicine receives minimal attention from both the pharmaceutical industry and academia as a consequence of the known link with toxicity and reactivity in humans. In addition, these substances were considered to have no clinical use in modern medicine.^49,51-53^ Since 1985, structurally diverse and defined organometallic compounds have been established in the field of organometallic chemistry, with studies focusing on the chemistry between at least one metal-carbon bond, while shedding light on the advantages of organometallic compounds and sharing insight on their role in metallopharmaceuticals/medicinal chemistry.^49,51,53,54^

The physico-chemical properties of organometallic compounds include being regularly uncharged, relatively lipophilic, having a low oxidation state from the metallic center, exhibiting chemical reactivity that is neither organic nor metallic, as seen in their single state, as well as the vast choice of metallic ions and ligands that could be paired.^52,54,55^ These are the reasons for the rekindled appeal of the use of these compounds in biological applications in the last two decades. This has also propelled the recognition of a more complex and nascent field of organometallic chemistry termed “bioorganometallic chemistry”, which introduces organometallic compounds in biomolecules such as proteins and nucleic acids.^49,54,56,57^

Bioorganometallic chemistry, which has significantly expanded the scope of structural diversity and molecular recognition, was only introduced in the 1980s but is now an extensive part of the development of various medical and biological fields. The potentially negative effects of bio-organometallic compounds that include factors pertaining to their stability in the presence of water and oxygen, their potential to interact with human DNA, as well the dangers of incorporating heavy transition metals in these compounds, continued to be investigated. Such studies aimed at elucidating these potentially deleterious relationships have had success, as shown in a recent study where the stability of these compounds in water and oxygen was confirmed.^49^ The use of bioorganometallic complexes, which have definitive metal-carbon bonds, has been expanded to serve as luminescent probes in imaging and radiopharmaceuticals, as anti-cancer, anti-bacterial and anti-parasitic agents, as well as in infra-red active tracers.^50,57^ In addition, the incorporation of biological macromolecules such as proteins and nucleic acids in organometallic structures similar to those found in the body, such as hemoglobin (with iron), creates opportunities of developing entirely new bio-active and biocidal organometallic compounds through the conjugation or modification of the macromolecule with an organometallic moiety.^56^ These metal complexes have also been used as sensors for proteins and to monitor protein-protein interactions.

## Bioorganometallic compounds in drug design

The success of bioorganometallic compounds in biology and medicine has driven research and development towards metallo-pharmaceuticals and the use of metallo-active species in drug discovery and design.^51^ The capacity of metal complexes in this regard offers the possibility to use electron transfer, substitution rates and reduction potentials in the tuning of ligand affinities, as well as the use of stable transition metals of variable and predictable structures such as secure preparations and most importantly, valuable information obtained on the use of metals in organisms and in biological targeting. These are criteria linked to success in their use to alleviate the burden of many devastating and globally important diseases including cancer, AIDS, malaria and other bacterial, viral and parasitic infections, as well as increasing the intensity/potency of established clinical drugs by tackling tolerance and resistance.^51,58^ In addition, the well-received success of bioorganometallic complexes in the treatment of such diseases has displayed its value in the development of drugs that are synthesizable, suitable for large-scale production, inexpensive for patients and have the potential to bypass drug resistance mechanisms.^48,59^ The launch of the initiative by the WHO to work towards combating the burden of NTDs incited reviews of the intrinsic nature of medicinal bioorganometallic compounds in new drug treatment solutions. Although not widely reported, bioorganometallic compounds used against NTDs such as African sleeping sickness (human African trypanosomiasis), Chagas disease, Leishmaniasis and schistosomiasis, have been recounted extensively and have registered good progress.^48,59,60^ Current literature includes studies employing metal complexes containing bioactive and non-bioactive ligands to inhibit various biosynthetic processes or disrupting redox homeostasis to investigate anti-trypanosomal activity, as well as studies reviewing the use of transition metal complexes such as gold and silver as anti-leishmanial candidates.^59^ Other precious metals such as platinum and ruthenium have also been linked to organometallic complexes that exhibit potential for therapeutic purposes.^38^

Another exciting and significant concept that metallo-therapeutic agents provide is the application of organometallic derivatization of old or known drugs. This approach, referred to as metal-drug synergism, involves designing compounds that are founded on the coordination of a transition of metal into organic compounds of known or potential biological activity.^51,58^ This not only increases the efficacy of the parent drug; it also introduces possibilities of synthesizing drugs with multiple targets against organisms that show drug resistance, making it difficult for them to develop, especially if resistance is linked to target modification.^58,61,62^ The phenomenon of metal-drug synergism has had the greatest success in anti-cancer (particularly ovarian and testicular cancer) and anti-malarial chemotherapeutic treatments.^53^ Exemplary work by exemplary work by Fish and Jaouen, as well as Biot et al on the anti-cancer drug, tamoxifen, and anti-malarial drug chloroquine in *Plasmodium* spp., which overcame resistance in chloroquine-resistant strains, is leading experimental studies to elucidate and achieve such relationships.^57,58^ Chemotherapeutic examples of these compounds have since received attention and have entered various clinical trials.^52,60^

## Bioorganometallic compounds against schistosomiasis

Schistosomiasis has a great global impact, burdening over 85% of people in SSA alone.^15,17^ With the emergence of resistance to the only chemotherapeutic drug available against the infectious disease, it is understandable why bioorganometallic chemists are examining metal complexes and metal-drug synergism as potentials for novel leads in anti-schistosome treatment.^54,63^ Although bioorganometallic derivatizations of PZQ, oxamniquine (OXA) and some repurposed anti-malarial and anti-fungal treatments have been created and tested, there is also immense potential in the ability of metal complexes to readily undergo electron transfer reactions that make them candidates for oxidative stress-induced drug targeting, particularly with the poorly adapted oxidative stress-coping mechanisms of schistosomes.^35,64^

### i. Oxamniquine

Oxamniquine is an anti-schistosomal drug primarily used against schistosomiasis caused by the *S. mansoni*species only.^6,11,65-67^ The tetrahydroquinoline compound^1^ was produced in Germany and the United Kingdom during the 1940s and is among a series of related drugs, such as hycanthone, which were not marketable owing to issues such as toxicity or mutagenicity.^67^ The ineffectiveness of the drug on *S. haematobium* and *S. japonicum* worms is a result of its mechanism of action, which requires the activity of sulfotransferase for conversion to its active form, an esterifying enzyme found only in the *S. mansoni* worm.^11,65,67^ Once in its active state as a sulfate ester, the ester dissociates in a non-enzymatic reaction to form electrophilic reactants capable of alkylating schistosomal DNA. This results in irreversible damage to nucleic acid metabolism, thereafter inhibiting protein synthesis and leading to a strikingly retarded death.^11,68,69^ Despite its effectiveness and extensive use in South American countries such as Brazil, the drug comes with its own set of associated side effects, which include sleep induction, occasional orange-red urine and epileptic seizures, as well as its reduced susceptibility to female worms. Moreover, the drug seems highly prone to resistance owing to alteration of the gene encoding the esterifying enzyme, resulting in it no longer being considered desirable in various countries.^6,11,69^ Another notable issue related to the lack of use of OXA in Africa is that many inexpensive alternatives have been mass-produced.^66^

In 2017, Hess and colleagues were able to derive metallocene compounds of OXA aimed specifically at the issues of species-specific effects of the drug and the possibility to modulate the drug’s properties, a technique for developing anti-parasitic compounds such chloroquine and monepantel. In this study, four ferrocenyl compounds that had different N-acylation and N-alkylationunderwent *in vitro*testing on juvenile and adult worms as well as* in vivo* testing done on *S. mansoni-*infected mice models. Incubation of the larval worms for 72 hours showed that two compounds were able to reach 100% and 76% worm killing respectively, while the other compounds both showed killing of <30%. Although these values exhibited greater reduction rates than OXA, which shows 67% effectiveness, mild toxicity was observed for all compounds in L6 rats skeletal myoblast cells after 72 hours, showing IC_50_ values of 57.9-100.3 µM; values that are close to that of the reference (IC_50_ > 90µM). In contrast, one of the compounds in the incubated *S. mansoni* adult worms showed potency against the worms after only 24 hours, while the rest of the compounds demonstrated mild cytotoxic effects, exhibiting worm viability of 24.3-40.5%.^70^

### ii. Anti-malarials and their derivatives

Despite single-celled malaria *Plasmodia* being phylogenetically distant from *Schistosoma* worms, various anti-malarial drugs have undergone testing, either alone or in combination with schistosome drugs, against schistosomiasis and this drug repurposing technique seems promising.^47,69^ This is mostly based on the blood-feeding similarity in the species, which both degrade hemoglobin into hemozoin, the key primary target in anti-plasmodial chemotherapy.^71^ Known to be part of a highly potent class of drugs used against malaria parasites, Artemisinin is the key ingredient extracted from the leaves of the *Artemisia annua* plant, which is endemic to countries such as China, Central Europe and Argentina.^11,66^ The drug is a sesquiterpene lactone with a peroxide bridge believed to be the active pharmacophore and from which various semi-synthetic compounds, including artemether, artesunate and artether, have been derived.^11,66,69^ Activity of these compounds against schistosome species was divulged in the early 1980s ^66^ and has been reported in a multitude of studies that include both human and animal experiments.^11^ The currently accepted hypothesis on its mechanism of action is that the drug functions through a heme-dependent reduction that subsequently results in the release of ROS, where the heme iron attacks and dissociates the active endoperoxide bridge to artemisinin to produce a carbon-free radical that results in lethal damage by alkylating parasite proteins^11,47^

In addition to being well tolerated and producing only mild side effects, artemisinin compounds, unlike OXA, show great activity against juvenile worms^66^ and are less effective against mature worms.^47^ This makes them suitable for combined treatment with the currently used PZQ, which is primarily active against adult worms.^11,47^ This has encouraged clinical use as prophylactics and their chemoprophylactic effect being demonstrated in randomized controlled trials conducted on *S. mansoni* and *S. haematobium* infections in Africa.^65,66^ Despite the proposed usefulness of artemisinin-based combined therapy in areas with high transmission rates, concerns have been raised about the possible emergence of artemisinin-resistant* Plasmodium* spp. particularly in areas in the SSA region, where malaria and schistosomiasis are co-endemic.^6,65,69^

#### Ferroquine

Ferroquine (FQ), the most industrially advanced organometallic compound, is derived from the anti-malarial drug chloroquine (CQ).^63^ The complex was generated through the addition of a ferrocenyl moiety on the organic drug in an effort to overcome chloroquine resistance in *Plasmodial*spp. The drug has novel and new modes of action from its parent drug that successfully bypass chloroquine-resistant parasitic strains. In 2014, Keiser and colleagues conducted i*n vivo*and *in vitro* screening of various chloroquine-derived compounds such as FQ, ruthenoquine (RQ) and hydroxyl ferroquine (FQ-OH) against newly transformed schistosomula (NTS) and adult *S. mansoni* worms. The two main mechanisms of action of the drug are inhibiting hemozoin formation and generating OH radicals in oxidizing conditions.^72^ FQ shares the same 4-aminoquinoline basic center with CQ that is responsible for its high lipophilicity and allows FQ to permeate membranes and accumulate in the digestive vacuole.^59,62^ FQ accumulation in the digestive vacuole prevents hemozoin biomineralization, which can cause cell death due to the lack of toxic heme products precipitating. The generation of OH radicals enables FQ to induce breakdown of the parasite digestive vacuole membrane, which is not seen in RQ or CQ. In addition, the OH radicals may be involved in the oxidation of glutathione, which is vital in the detoxification of heme. In the study, it was observed that FQ-OH also produces radicals but has a reduced cytotoxic effect compared to FQ. Although all compounds showed moderate activity against larval and adult *S. mansoni*worms after 72 hours’ incubation *in vitro*, none displayed any activity during *in vivo* screening. *S. mansoni*-infected mice showed low total worm reductions of 0-36% during observation following oral administration of 200-800 mg/kg of the FQ derivatives. All the metallocenes exhibited moderate cytotoxicity towards MRC-5 mammalian cells, while higher toxicity levels were observed towards HeLa cancer cells.

#### Tetraazamacrocyclic derivatives and their metal complexes

Macrocyclic polyamines, such as triazacyclononane, cyclam and cyclen, are a special group of heterocycles with various biological and chemical applications due to their importance as ligands of ionic complexes.^73^ Macrocycles have gained popularity as structural moieties used in various diagnostic and therapeutic pharmaceutical agents.^74^ The activity of synthetic macrocyclic polyamines and their metal complexes has been observed against HIV and through metal ion coordination, while metal complexes containing tetraazamacrocyclic ligands have shown potential as anti-malarial agents.^75-77^ In 2016, Khan et al extended their library of synthetic cross- and side-bridged tetraazamacrocyclic ligands used in their anti-malarial and anti-HIV drug discovery program towards anti-schistosomal drug research. The study involved phased screening of 26 tetraazamacrocyclic derivatives, seven of which were newly described, as well as with their metal complexes for anti-helminthic activity against *S. mansoni in vivo* and *in vitro.*The compounds were initially screened against NTS from harvested *S. mansoni* cercariae and adult worms, and finally during *in vivo* testing of *S. mansoni*-infected mouse models. Incubation of 12 compounds with a concentration of 33 µM resulted in a 62-100% mortality level of NTS. Five compounds exhibiting 100% inhibition of viability of NTS at 10 µM were further screened for IC_50_ values against both NTS and adult worms. All compounds screened against NTS showed IC_50_ values that were comparable to values observed with the standard PZQ. In addition, three of the five compounds, which were bisquinoline derivatives of cyclen, and their Fe^2+^ and Mn^2+^ complexes showed only micromolar IC_50_ values against adult worms. *In vivo* screening showed worm burden reductions of 12.3%, 74.5% and 88.4% respectively, after a single oral dose of 400 mg/kg. A possible novel drug lead for schistosomiasis that is comparable to the standard PZQ drug was hence discovered in a Fe^2+^ complex that exhibited activity *in vivo.*^61^

### iii. Praziquantel

Praziquantel is the generic name for (2-cyclohexylcarbonyl)-1,2,3,6,7,11b-hexa-hydro-4H-pyrazino(2,1-α) isoquinoline-4-one, a bitter-tasting white crystalline powder created in the pyrazino-isoquinoline ring system, which was initially created for the development of a tranquilizer compound.^11,66,68^ After the anti-helminthic properties of the compound were discovered in the mid-1970s against all schistosome species and cestodes, the drug gained great momentum, becoming the cornerstone of all schistosome treatments.^11,68^ The compound, which is now marketed as Biltricide^®^, is stable under normal storage conditions, essentially insoluble in water, but sparingly soluble in ethanol and soluble in organic solvents.^11,68^ The drug, which has now been in use for over 40 years, is marketed as 600 mg tablets with a standard dose of 40 mg/kg for *S. haematobium* and *S. mansoni*infections, while a 60 mg/kg dose is recommended for *S. japonicum* and *S. mekongi* infections.^1^ The main contributing factors to PZQ being described as the best mono-therapeutic agent against schistosomiasis can be attributed to its inexpensiveness, safety, high efficacy, accessibility and effectiveness against all human schistosome species.^11,67^ In addition, the drug only exhibits limited and mild side effects such as vomiting and diarrhea.

#### PZQ mode of action

The physiological and morphological effects of PZQ primarily include three main actions: (1) the rapid influx of Ca^2+^ions, (2) vacuolation and blebbing of adult schistosome worm teguments and (3) muscle contraction.^66,68,78^ However, the precise mechanism leading to the schistosomicidal effect of the drug is yet to be elucidated.^5,11,66,79^ A well-supported hypothesis is a link between the increase in intracellular Ca^2+^ions, which is usually followed by a slow influx of sodium and potassium ions^5^, and the resulting muscle contraction and alteration of the worm tegument. Although the exact identity and location of the molecular target responsible for the disruption of the calcium homeostasis is still unknown, this phenomenon exposes worm surface antigens, enabling recognition by host immune system defenses.^78-80^ For this reason, it was hypothesized in various studies, such as those of Kohn et al 2001 and 2003^81,82^ and Jeziorski and Greenberg,^83^ that voltage-operated calcium channels (VOCC) are responsible for calcium accumulation.^65,78^ This molecular target, which could be any of various cellular factors that regulate intracellular calcium, is thought to function primarily on the two β-subunits on the VOCC found in *S. mansoni* and *S. japonicum* species, which indicate differences in structural and functional motifs to conventional β-subunits.^5,11,66,79,83^ The subunits, a homologous form that is common in mammals and a variant type that lacks two conserved serine residues associated with a consensus site for protein kinase C phosphorylation, result in schistosome sensitivity to PZQ.^66,78^

#### PZQ reduced susceptibility and resistance

The mono-therapeutic use of PZQ has raised many concerns in the scientific community. The factors of concern are primarily the stage-dependency of the drug and the lack of efficacy on juvenile schistosome worms, which could be linked to the poor rate of cures and treatment failures in residents of high-risk regions.^66^ The most significant shortcoming of the drug that is being debated is its inclination to resistance and its reduced susceptibility towards schistosome worms, as shown in various field and laboratory-based experiments.^78^ In addition, the undetermined nature of the mechanism of action of the drug not only makes it difficult to derive compounds focusing on the same molecular target; it becomes even harder to determine the possible mechanism of resistance.^66^ Reviewing the evidence of the results from both experimental settings requires consideration of possible genetic, physiological and morphological markers to indicate possible resistance.^67,79^ Studies that attempted experimental induction of PZQ resistance began in the 1970s and have continued to modern times with the use of lethal to sub-lethal doses of the drug, with great attention being paid to *S. mansoni* species.^78,79^ Success in the selection of laboratory-induced PZQ-resistant *S. mansoni* worms was then reported in 1994 by Fallon and Doenhoff.^67,84^ The phenomenon was again observed in two separate studies by Liang et al^85^ and Pica-Mattoccia and Cioli.^86^ A different study conducted by Ismail et al^87^ found that the use of low sub-curative doses of PZQ may lead to the development of PZQ resistance in subsequent generations, which could be attributed to host factors as well as the pathogen itself. This study has recently led to a faster, simpler and far cheaper technique to produce laboratory-induced PZQ-resistant *S. mansoni* worms by successive treatment of *Biomphalaria glabrata* snails with the worms^5,84,88^ Apart from experimentally induced PZQ-resistant isolates, some field evidence has been reported in *S. mansoni*isolates of many endemic foci of African countries.^66,89^ Evidence from well-observed *in vivo* and *in vitro* assays of a KCW isolate obtained from a patient in Kisumu, Kenya showed miracadia that were tolerant to PZQ. In addition, similar observations on adult worms exhibited alarmingly low susceptibility to the drug compared to other adult Kenyan isolates and laboratory stocks. The isolates maintained heritable resistance that persisted over six generations.^5,90^

#### PZQ-derived organometallic compounds

Praziquantel has been used to derive various structural variations of non-resistant organometallic analogues.^91^ Chemical modifications of PZQ using organometallic moieties are a possible approach to overcoming the issues and drawbacks of the drug. Therefore, various authors^60,92^ have studied the potential relationship of such organometallic moieties with PZQ and although no compelling compounds have gone to clinical testing and trials, some of these studies have been able to bring about insight into approaching this technique, as seen in a report by Patra et al, whose studies concluded that the C10 aromatic ring of PZQ is not suitable for structural modification.^60^

A study conducted by Hess and and colleagues between 2012 and 2013 studied three strategies to modify PZQ. These strategies were primarily based on the addition of a ferrocene moiety similar to FQ (a chloroquine derivative), which has successfully evaded and increased resistance to the drug.^54^ In the study, strategies I and II were aimed at modifying the cyclohexyl moiety, while strategy III was aimed at modifying the aromatic ring.^59^ Compounds lacking the cyclohexane ring can avoid the transformation to a hydroxylated metabolite with decreased activity, while those focusing on the aromatic ring have been shown not to affect the overall schistosomicidal effect of the compounds.^54^

With that information, a different study carried out by Patra and colleagues focused on the cyclohexane of PZQ and replaced it with a ferrocene-based unit. In the study, 18 ferrocenyl-derived PZQ compounds were synthesized as a racemic mixture and their activity was studied. The study was able to indicate the stability of these types of compounds in human plasma by using the LS-MS technique.^60^ The study made use of the MRC-5 and HeLa cervical cancer mammalian cell lines. In a selective evaluation, all 18 organometallic compounds lacked potency towards healthy MRC-5 fibroblast cells. Results also showed that the compounds were non-hazardous towards these cells but exhibited mild-moderate to cytotoxic effects towards a cervical cancer cell line (HeLa), showing IC_50_ values ranging from 16.9-97.7 µM. In addition*, in vitro*screening of these compounds indicated that four active compounds had IC_50_ values that were significantly greater than that of PZQ (0,1 µM) at 25.6-68 µM.

Subsequent studies were focused on strategy III and included diastereomeric derivatives of chromium-tricarbonyl-PZQ synthesized in a one-step heat reaction of PZQ-[Cr(CO)_6_] at 140°C. The chromium-based derivatives were chosen for their water- and air-stable compounds and because chromium moieties are able to increase lipophilicity and metabolic stability and have the potential to alter the structural and electronic properties of the PZQ aromatic ring. The Cr-PZQ racemates were screened on *S. mansoni* worms and showed IC_50_ values of 0.25 and 0.27 µM, which are highly comparable to that of PZQ alone. These compounds also showed no toxicity towards MRC-5 human fibroblast cells, thus exhibiting some selective anti-parasitic effect. This preliminary result encouraged two separate studies on these compounds, one aimed at elucidating the activity or role of enantiomers in the activity of PZQ against schistosomes and the second studying the imaging of one of the compounds within the worm. The former was able to conclude that the R-enantiomer of PZQ showed anti-schistosomal activity, while the S-enantiomer could not, a possible indication of activity of the enantiomer of the Cr-PZQ compound. The second study was undertaken to determine the mechanism of action of the entire compound. Regardless of minimal knowledge of the mode of action of the parent drug, the hypothesis that its biological function is on the tegumental phospholipids was supported in this study through mass spectroscopic techniques where spectro-microscopy and X-ray fluorescence were used in combination to determine the exact location of one of the compounds. Signatures of the CO (IR) and Cr (XRF) showed the accumulation of the compound in the parasite tegument.^59^

### iv. Epiisopiloturine-metal complexes

Epiisopiloturine (EPI) is an imidazole alkaloid obtained as a secondary by-product from the production of pilocarpine, a compound extracted from the leaves of the *Pilocarpusmicrophyllus*plant native to the Amazon regions of Brazil.^59^ After purification and characterization, the possible schistosomicidal activity of the drug was described after noticeable tegumental alteration and death of adult worms had been verified at a concentration of 150 µg/mL, while the death of schistosomula was observed within 120 hours of 300 µg/mL being administered. In a study by Portes et al, EPI was complexed with copper (II) and zinc (II) salts with the aim of improving its anti-parasitic properties through the coordination of metal ions and the corresponding metal complexes were investigated for anti-helminthic activity.^93^ The study found that while coordination with copper (II) enhanced the activity of free EPI, coordination with zinc (II) decreased its activity. This led to the speculation that copper acted as a carrier of the drug within the parasite, causing a synergistic effect, and that the zinc-EPI complex had no anti-helminthic activity. Although no IC_50_ values were provided in the study, none of the complexes showed toxicity towards mammalian cells and the overall anti-schistosomal activity of the complexes was measured on tegumental modifications, egg-laying ability, and the reduction of motor mobility and mortality.^59^ Approximately 60% of tegumental disruptions such as sloughing was observed after 24-hour incubation periods with 250 µM of [Cu(EPI)_4_](ClO_4_)_2_. Significant oviposition was also observed in both complexes in the 60-250 µM range, with major egg-laying reduction of below 25% at a 62.5 µM concentration of the Cu-EPI complex. The high bioactivity of the copper complex is proposed to be due to the redox activity of copper, although other mechanisms of action independent of ROS generation cannot be ruled out.

## Redox-based organometallic compounds in anti-schistosome drugs

The survival of parasites is strongly linked to the redox system.^38^ The vulnerability of the schistosome worm to reactive species from both their aerobic metabolism and human host leukocytes as well as the limited capacity of their pro-oxidant defense mechanisms makes them attractive targets for anti-parasitic drugs. The versatility and tunability of redox-active metals in the synthesis of organometallic compounds creates an abundant range of anti-infective possibilities. The synthesis of bio-organometallic compounds with a redox center or the ability to affect redox biology resulting in parasite death is promising, since it has been described as the parasite’s Achilles heel.^28^ The activity of redox-based drug compounds could be aimed at three groups: (i) compounds that are aimed at inhibiting enzyme-catalyzed redox balance activities, (ii) preventing the scavenging of pro-oxidant metabolic products, and (iii) synthesizing molecules that are capable of generating ROS themselves and causing parasite death.^38^

### i. Auranofin

The recent discovery of the multifunctional selenoenzyme, TGR in *S. mansoni*and its importance in the cellular redox systems of the worm has made it one of the key candidates for the development of novel drugs.^28,36^ The gold-containing compound auranofin (AF), which has been used clinically as an anti-arthritic drug, has been shown to be a potential inhibitor of TGR.^59^ The study, which was conducted in 2009, showed TGR inhibition by AF and led to substantial reduction of the worm burden in mice. A concentration of 10 µM AF after 1 hour incubation *in vitro* caused unpairing of male and female worms and 100% mortality after 9 hours exposure. Furthermore, 5 µM AF incapacitated the larval, juvenile and adult stages of the parasite after 24 hours. This was significant, as this concentration of the drug has been proven through toxicity profiling to be tolerable in mammalian cells. Preliminary experiments on infected mice treated with AF showed tolerance by the host, killing over 60% of adult schistosome worms. This significant worm burden reduction is speculated to result in a significant plunge in the pathology and morbidity linked to the parasitic infection. Through IC_50_ values, AF demonstrated a nanomolar inhibitor of selenocysteine (sec)-containing enzymes and required over a 1000-fold higher concentration for enzymes lacking sec.

## Redox-directed potential of bioorganometallic compounds in schistosomiasis treatment

**Figure 1:**
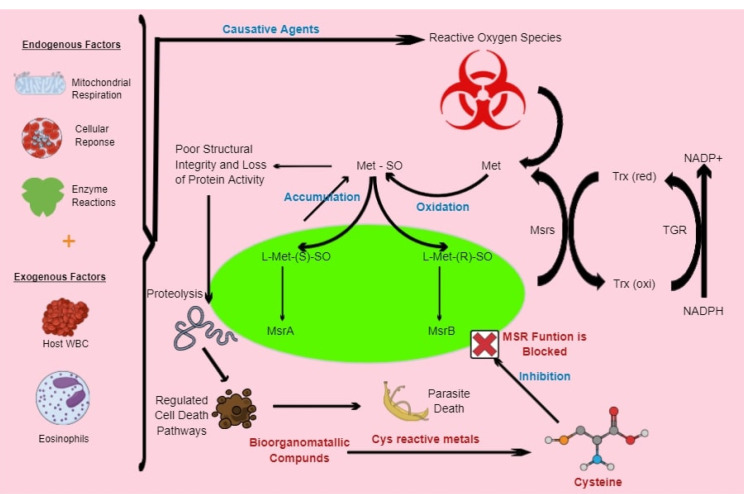
Model presenting potential of bioorganometallic compounds against schistosome Msrs. The oxidation-sensitive, sulfur-containing cysteine (cys) residue on the proteins’ active site can be used to inhibit Msrs through cys-reactive metals in bioorganometallic compounds, which will result in the accumulation of free and peptide-bound Met-SO. These reactive Met-SO residues will result in the alteration of structural integrity, conformational changes and decreased protein activity. The resulting protein damage will induce proteolysis and cell death pathways that can ultimately result in parasite death.

**Figure 2:**
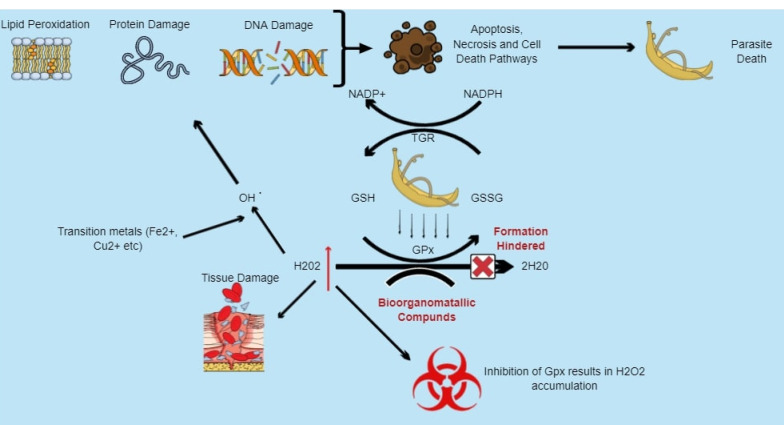
Model presenting potential of bioorganometallic compounds against schistosome glutathione peroxidase (GPx). Hydrogen peroxide (H_2_O_2_) within schistosomes is reduced to water (H_2_O) by the enzyme GPx as they lack catalase. The enzyme, similar to Msrs, contains cysteine residues within its active site that are susceptible to spontaneous oxidation and can be inhibited using cys-reactive bioorganometallic compounds, inhibiting the conversion of H_2_O_2_ and leading to its accumulation, which results in oxidative stress that can cause lipid membrane peroxidation, DNA strand breakage, base-sequence gaps, as well as protein damage.

**Figure 3:**
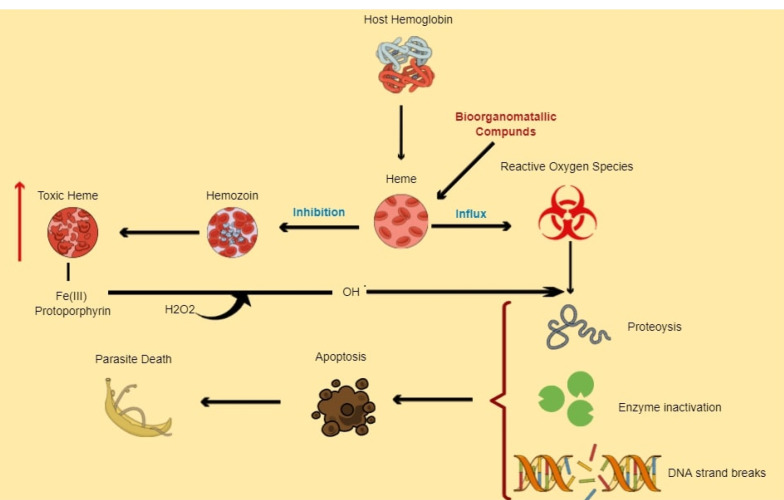
Model presenting potential of bioorganometallic compounds in heme detoxifying pathway. Bioorganometallic compounds that can bind and react with toxic heme (Fe (III) protoporphyrin) can create a bioorganometallic-heme complex that hinders hemozoin formation, resulting in toxic heme accumulation, which enhances oxidative stress. The increasing Fe (III) protoporphyrin can also react with schistosome hydrogen peroxide to form more reactive radicals that can collectively cause cytotoxic damage to schistosome DNA, proteins and lipids that can in turn result in an increase in cell death pathways, ultimately causing parasite death.

The unique and vulnerable redox biology of schistosomes provides several targets for the development of novel anti-schistosomal agents.^28^ Inhibiting schistosome anti-oxidant enzymes such as Msrs (Figure 1), GPx (Figure 2) and their various associated redox reactions using bioorganometallic compounds hinders their ROS scavenging activity, presenting an opportunity for oxidative stress-induced parasite tissue damage caused by accumulating Met-SO and H_2_O_2_ levels respectively. In addition, proteolytic digestion of host haemoglobin results in the formation of free heme, which the worm detoxifies through aggregation into the inert crystalline polymer, hemozoin. Inhibition of heme-detoxifying pathways using bioorganometallic compounds can result in its accumulation, inducing oxygen-derived free radical formation, lipid peroxidation, loss of enzyme activity and cell lysis mechanisms (Figure 3).^28,94,95^ The oxidation of proteins, which involves protein carbonylation, oxidation-induced cleavage or conjugation of lipid peroxidation products, increases protein hydrophobicity, thus affecting cell signaling, cell structure and enzymatic reactions, including metabolic processes.^96,97^ The oxygen-rich environment also poses a threat to parasite DNA, which becomes susceptible to mutations, deletions and breaks in DNA links that cannot be rectified owing to loss of enzyme activity.^98,99^ Oxidative stress also causes peroxidation of the polyunsaturated fatty acids, initiating a series of self-propagating reactions that destroy membrane lipids, generate highly dangerous end-products that disrupt cell viability and can cross-link with other macromolecules such as proteins.^100,101^ These damaging cell and tissue patterns result in non-physiological (necrotic) and regulated pathways (apoptosis) that can ultimately result in parasite death.^10^

## Conclusion

The most significant burden of parasitic infections is experienced in developing countries where poverty and poor hygienic practices have detrimental health consequences. Alongside malaria, schistosomiasis is one the greatest challenges in the SSA region. Various control and elimination interventions that range from the use of molluscicides to mass drug administration of different chemotherapeutic agents have been implemented with different success rates. Praziquantel has been the most successful in reducing morbidity and mortality rates, becoming the standard drug against the disease. Despite this, issues related to the emergence of drug resistance incited the application of bioorganometallic compounds against the weak redox biology of schistosomes. These metal-based, redox-directed compounds have the potential to develop new mechanisms of action that can bypass drug resistance, overcoming and possibly leading research towards eliminating this debilitating infection.

## Ethical Issues

Not applicable.

## Conflict of Interest

None declared.

## References

[1] Colley DG, Bustinduy AL, Secor WE, King CH (2014). Human schistosomiasis. Lancet.

[2] Costain AH, MacDonald AS, Smits HH (2018). Schistosome egg migration: mechanisms, pathogenesis and host immune responses. Front Immunol.

[3] Zhou L, Yu L, Wang Y, Lu Z, Tian L, Tan L (2014). A hybrid model for predicting the prevalence of schistosomiasis in humans of Qianjiang city, China. PLoS One.

[4] Ishida K, Jolly ER (2016). Hsp70 may be a molecular regulator of schistosome host invasion. PLoS Negl Trop Dis.

[5] Masamba P, Adenowo AF, Oyinloye BE, Kappo AP (2016). Universal stress proteins as new targets for environmental and therapeutic interventions of schistosomiasis. Int J Environ Res Public Health.

[6] Gryseels B, Polman K, Clerinx J, Kestens L (2006). Human schistosomiasis. Lancet.

[7] Chitsulo L, Engels D, Montresor A, Savioli L (2000). The global status of schistosomiasis and its control. Acta Trop.

[8] Grimes JE, Croll D, Harrison WE, Utzinger J, Freeman MC, Templeton MR (2015). The roles of water, sanitation and hygiene in reducing schistosomiasis: a review. Parasit Vectors.

[9] Hotez PJ, Brindley PJ, Bethony JM, King CH, Pearce EJ, Jacobson J (2008). Helminth infections: the great neglected tropical diseases. J Clin Invest.

[10] Nour NM (2010). Schistosomiasis: health effects on women. Rev Obstet Gynecol.

[11] Aruleba RT, Adekiya TA, Oyinloye BE, Masamba P, Mbatha LS, Pretorius A (2019). PZQ therapy: how close are we in the development of effective alternative anti-schistosomal drugs?. Infect Disord Drug Targets.

[12] Hotez PJ, Kamath A (2009). Neglected tropical diseases in sub-Saharan Africa: review of their prevalence, distribution, and disease burden. PLoS Negl Trop Dis.

[13] Adenowo AF, Oyinloye BE, Ogunyinka BI, Kappo AP (2015). Impact of human schistosomiasis in sub-Saharan Africa. Braz J Infect Dis.

[14] Otuneme OG, Obebe OO, Sajobi TT, Akinleye WA, Faloye TG (2019). Prevalence of schistosomiasis in a neglected community, South western Nigeria at two points in time, spaced three years apart. Afr Health Sci.

[15] Sacolo-Gwebu H, Kabuyaya M, Chimbari M (2019). Knowledge, attitudes and practices on schistosomiasis and soil-transmitted helminths among caregivers in Ingwavuma area in uMkhanyakude district, South Africa. BMC Infect Dis.

[16] van der Werf MJ, de Vlas SJ, Brooker S, Looman CW, Nagelkerke NJ, Habbema JD (2003). Quantification of clinical morbidity associated with schistosome infection in sub-Saharan Africa. Acta Trop.

[17] Tchuem Tchuenté LA, Rollinson D, Stothard JR, Molyneux D (2017). Moving from control to elimination of schistosomiasis in sub-Saharan Africa: time to change and adapt strategies. Infect Dis Poverty.

[18] Conteh L, Engels T, Molyneux DH (2010). Socioeconomic aspects of neglected tropical diseases. Lancet.

[19] Morgan JA, Dejong RJ, Snyder SD, Mkoji GM, Loker ES (2001). Schistosoma mansoni and Biomphalaria: past history and future trends. Parasitology.

[20] Blanton RE (2019). Population structure and dynamics of helminthic infection: schistosomiasis. Microbiol Spectr.

[21] Oliveira MP, Correa Soares JB, Oliveira MF (2016). Sexual preferences in nutrient utilization regulate oxygen consumption and reactive oxygen species generation in Schistosoma mansoni: potential implications for parasite redox biology. PLoS One.

[22] Mostafa MH, Sheweita SA, O’Connor PJ (1999). Relationship between schistosomiasis and bladder cancer. Clin Microbiol Rev.

[23] Zaghloul MS (2012). Bladder cancer and schistosomiasis. J Egypt Natl Canc Inst.

[24] Herman AM, Kishe A, Babu H, Shilanaiman H, Tarmohamed M, Lodhia J (2017). Colorectal cancer in a patient with intestinal schistosomiasis: a case report from Kilimanjaro Christian Medical Center Northern Zone Tanzania. World J Surg Oncol.

[25] Madbouly KM, Senagore AJ, Mukerjee A, Hussien AM, Shehata MA, Navine P (2007). Colorectal cancer in a population with endemic Schistosoma mansoni: is this an at-risk population?. Int J Colorectal Dis.

[26] The Lancet (2019). The LancetThe L2020: a crucial year for neglected tropical diseases. Lancet.

[27] Oyinloye BE, Adenowo AF, Kappo AP (2015). Reactive oxygen species, apoptosis, antimicrobial peptides and human inflammatory diseases. Pharmaceuticals (Basel).

[28] Huang HH, Rigouin C, Williams DL (2012). The redox biology of schistosome parasites and applications for drug development. Curr Pharm Des.

[29] Valko M, Morris H, Cronin MT (2005). Metals, toxicity and oxidative stress. Curr Med Chem.

[30] Reniere ML (2018). Reduce, induce, thrive: bacterial redox sensing during pathogenesis. J Bacteriol.

[31] Trachootham D, Lu W, Ogasawara MA, Nilsa RD, Huang P (2008). Redox regulation of cell survival. Antioxid Redox Signal.

[32] Sharma M, Khanna S, Bulusu G, Mitra A (2009). Comparative modeling of thioredoxin glutathione reductase from Schistosoma mansoni: a multifunctional target for antischistosomal therapy. J Mol Graph Model.

[33] Johann L, Lanfranchi DA, Davioud-Charvet E, Elhabiri M (2012). A physico-biochemical study on potential redox-cyclers as antimalarial and anti-schistosomal drugs. Curr Pharm Des.

[34] Panic G, Duthaler U, Speich B, Keiser J (2014). Repurposing drugs for the treatment and control of helminth infections. Int J Parasitol Drugs Drug Resist.

[35] Sayed AA, Cook SK, Williams DL (2006). Redox balance mechanisms in Schistosoma mansoni rely on peroxiredoxins and albumin and implicate peroxiredoxins as novel drug targets. J Biol Chem.

[36] Kuntz AN, Davioud-Charvet E, Sayed AA, Califf LL, Dessolin J, Arnér ES (2007). Thioredoxin glutathione reductase from Schistosoma mansoni: an essential parasite enzyme and a key drug target. PLoS Med.

[37] Oke TT, Moskovitz J, Williams DL (2009). Characterization of the methionine sulfoxide reductases of Schistosoma mansoni. J Parasitol.

[38] Pal C, Bandyopadhyay U (2012). Redox-active antiparasitic drugs. Antioxid Redox Signal.

[39] Salinas G, Selkirk ME, Chalar C, Maizels RM, Fernández C (2004). Linked thioredoxin-glutathione systems in platyhelminths. Trends Parasitol.

[40] Song L, Li J, Xie S, Qian C, Wang J, Zhang W (2012). Thioredoxin glutathione reductase as a novel drug target: evidence from Schistosoma japonicum. PLoS One.

[41] Tripathi T, Suttiprapa S, Sripa B (2017). Unusual thiol-based redox metabolism of parasitic flukes. Parasitol Int.

[42] Kim HY (2013). The methionine sulfoxide reduction system: selenium utilization and methionine sulfoxide reductase enzymes and their functions. Antioxid Redox Signal.

[43] Chigor VN, Eze EA, Olasan JO, Yandev D, Ishwua MN, Onwuka AU (2019). Socioeconomic factors associated with high prevalence of schistosomiasis among rural dwellers in Benue State, Nigeria. Int J Innov Res Adv Stud.

[44] Uneke CJ (2010). Soil transmitted helminth infections and schistosomiasis in school age children in sub-Saharan Africa: efficacy of chemotherapeutic intervention since World Health Assembly Resolution 2001. Tanzan J Health Res.

[45] Lai YS, Biedermann P, Ekpo UF, Garba A, Mathieu E, Midzi N (2015). Spatial distribution of schistosomiasis and treatment needs in sub-Saharan Africa: a systematic review and geostatistical analysis. Lancet Infect Dis.

[46] Stothard JR, Chitsulo L, Kristensen TK, Utzinger J (2009). Control of schistosomiasis in sub-Saharan Africa: progress made, new opportunities and remaining challenges. Parasitology.

[47] Bergquist R, Elmorshedy H (2018). Artemether and praziquantel: origin, mode of action, impact, and suggested application for effective control of human schistosomiasis. Trop Med Infect Dis.

[48] Brown RW, Hyland CJT (2015). Medicinal organometallic chemistry–an emerging strategy for the treatment of neglected tropical diseases. MedChemComm.

[49] Coogan MP, Dyson PJ, Bochmann M (2012). Introduction to the organometallics in biology and medicine issue. Organometallics.

[50] Kowalski K, Hikisz P, Szczupak Ł, Therrien B, Koceva-Chyła A (2014). Ferrocenyl and dicobalt hexacarbonyl chromones--new organometallics inducing oxidative stress and arresting human cancer cells in G2/M phase. Eur J Med Chem.

[51] Eke UB, Abubakar TA (2015). Bioorganometallic compounds in medicine: the search for new antibacterial agents. World J Biomed Pharm Sci.

[52] Gasser G, Metzler-Nolte N (2012). The potential of organometallic complexes in medicinal chemistry. Curr Opin Chem Biol.

[53] Navarro M, Visbal G. Metal-based antiparasitic therapeutics. In: Nriagu JO, Skaar EP, eds. Trace Metals and Infectious Diseases. Cambridge: MIT Press; 2016. p. 161-72. 10.7551/mitpress/9780262029193.003.0010

[54] Hess J, Keiser J, Gasser G (2015). Toward organometallic antischistosomal drug candidates. Future Med Chem.

[55] Dougan SJ, Habtemariam A, McHale SE, Parsons S, Sadler PJ (2008). Catalytic organometallic anticancer complexes. Proc Natl Acad Sci U S A.

[56] Carraher CE Jr, Pittman CU Jr. Organometallic compounds in biomedical applications. In: Abd-El-Aziz AS, Carraher CE Jr, Pittman CU Jr, Sheats JE, Zeldin M, eds. Macromolecules Containing Metal and Metal-Like Elements. Vol 3. John Wiley & Sons; 2004. p. 1-18. 10.1002/0471683779.ch1

[57] Fish RH, Jaouen G (2003). Bioorganometallic chemistry: structural diversity of organometallic complexes with bioligands and molecular recognition studies of several supramolecular hosts with biomolecules, alkali-metal ions, and organometallic pharmaceuticals. Organometallics.

[58] Biot C, Castro W, Botté CY, Navarro M (2012). The therapeutic potential of metal-based antimalarial agents: implications for the mechanism of action. Dalton Trans.

[59] Ong YC, Roy S, Andrews PC, Gasser G (2019). Metal compounds against neglected tropical diseases. Chem Rev.

[60] Patra M, Ingram K, Pierroz V, Ferrari S, Spingler B, Keiser J (2012). Ferrocenyl derivatives of the anthelmintic praziquantel: design, synthesis, and biological evaluation. J Med Chem.

[61] Khan MO, Keiser J, Amoyaw PN, Hossain MF, Vargas M, Le JG (2016). Discovery of antischistosomal drug leads based on tetraazamacrocyclic derivatives and their metal complexes. Antimicrob Agents Chemother.

[62] Patra M, Gasser G, Metzler-Nolte N (2012). Small organometallic compounds as antibacterial agents. Dalton Trans.

[63] Gasser G (2015). Metal complexes and medicine: a successful combination. Chimia (Aarau).

[64] Sotillo J, Pearson M, Becker L, Mulvenna J, Loukas A (2015). A quantitative proteomic analysis of the tegumental proteins from Schistosoma mansoni schistosomula reveals novel potential therapeutic targets. Int J Parasitol.

[65] Doenhoff MJ, Hagan P, Cioli D, Southgate V, Pica-Mattoccia L, Botros S (2009). Praziquantel: its use in control of schistosomiasis in sub-Saharan Africa and current research needs. Parasitology.

[66] Doenhoff MJ, Cioli D, Utzinger J (2008). Praziquantel: mechanisms of action, resistance and new derivatives for schistosomiasis. Curr Opin Infect Dis.

[67] Doenhoff MJ, Wheatcroft-Francklow K. Schistosome drug resistance. In: Gillespie SH, ed. Management of Multiple Drug-Resistant Infections. Totowa, NJ: Humana Press; 2004. p. 341-52. 10.1007/978-1-59259-738-3_19

[68] Cioli D, Pica-Mattoccia L, Archer S (1995). Antischistosomal drugs: past, present and future?. Pharmacol Ther.

[69] Gouveia MJ, Brindley PJ, Gärtner F, Costa J, Vale N (2018). Drug repurposing for schistosomiasis: combinations of drugs or biomolecules. Pharmaceuticals (Basel).

[70] Hess J, Panic G, Patra M, Mastrobuoni L, Spingler B, Roy S (2017). Ferrocenyl, ruthenocenyl, and benzyl oxamniquine derivatives with cross-species activity against Schistosoma mansoni and Schistosoma haematobium. ACS Infect Dis.

[71] Keiser J, N’Guessan NA, Adoubryn KD, Silué KD, Vounatsou P, Hatz C (2010). Efficacy and safety of mefloquine, artesunate, mefloquine-artesunate, and praziquantel against Schistosoma haematobium: randomized, exploratory open-label trial. Clin Infect Dis.

[72] Keiser J, Vargas M, Rubbiani R, Gasser G, Biot C (2014). In vitro and in vivo antischistosomal activity of ferroquine derivatives. Parasit Vectors.

[73] Liang F, Wan S, Li Z, Xiong X, Yang L, Zhou X (2006). Medical applications of macrocyclic polyamines. Curr Med Chem.

[74] Baker WC, Choi MJ, Hill DC, Thompson JL, Petillo PA (1999). Synthesis of lipophilic paramagnetic contrast agents. J Org Chem.

[75] Khan A, Nicholson G, McRobbie G, Greenman J, Pannecouque C, Daelemans D (2009). CXCR4 antagonists: a new generation of configurationally restricted bis-azamacrocyclic compounds. Antiviral Res.

[76] Hubin TJ, Amoyaw PN, Roewe KD, Simpson NC, Maples RD, Carder Freeman TN (2014). Synthesis and antimalarial activity of metal complexes of cross-bridged tetraazamacrocyclic ligands. Bioorg Med Chem.

[77] Khan MO, Levi MS, Tekwani BL, Khan SI, Kimura E, Borne RF (2009). Synthesis and antimalarial activities of cyclen 4-aminoquinoline analogs. Antimicrob Agents Chemother.

[78] Vale N, Gouveia MJ, Rinaldi G, Brindley PJ, Gärtner F, Correia da Costa JM (2017). Praziquantel for schistosomiasis: single-drug metabolism revisited, mode of action, and resistance. Antimicrob Agents Chemother.

[79] Cupit PM, Cunningham C (2015). What is the mechanism of action of praziquantel and how might resistance strike?. Future Med Chem.

[80] Greenberg RM (2005). Are Ca2+ channels targets of praziquantel action?. Int J Parasitol.

[81] Kohn AB, Roberts-Misterly JM, Anderson PA, Khan N, Greenberg RM (2003). Specific sites in the Beta Interaction Domain of a schistosome Ca2+ channel beta subunit are key to its role in sensitivity to the anti-schistosomal drug praziquantel. Parasitology.

[82] Kohn AB, Anderson PA, Roberts-Misterly JM, Greenberg RM (2001). Schistosome calcium channel beta subunits Unusual modulatory effects and potential role in the action of the antischistosomal drug praziquantel. J Biol Chem.

[83] Jeziorski MC, Greenberg RM (2006). Voltage-gated calcium channel subunits from platyhelminths: potential role in praziquantel action. Int J Parasitol.

[84] Wang W, Wang L, Liang YS (2012). Susceptibility or resistance of praziquantel in human schistosomiasis: a review. Parasitol Res.

[85] Liang YS, Coles GC, Doenhoff MJ, Southgate VR (2001). In vitro responses of praziquantel-resistant and -susceptible Schistosoma mansoni to praziquantel. Int J Parasitol.

[86] Pica-Mattoccia L, Cioli D (2004). Sex- and stage-related sensitivity of Schistosoma mansoni to in vivo and in vitro praziquantel treatment. Int J Parasitol.

[87] Ismail M, Botros S, Metwally A, William S, Farghally A, Tao LF (1999). Resistance to praziquantel: direct evidence from Schistosoma mansoni isolated from Egyptian villagers. Am J Trop Med Hyg.

[88] Couto FF, Coelho PM, Araújo N, Kusel JR, Katz N, Jannotti-Passos LK (2011). Schistosoma mansoni: a method for inducing resistance to praziquantel using infected Biomphalaria glabrata snails. Mem Inst Oswaldo Cruz.

[89] Qian K, Liang Y, Wang W, Qu G, Li H, Yang Z (2018). Effect of praziquantel treatment on hepatic egg granulomas in mice infected with praziquantel-susceptible and resistance Schistosoma japonicum isolates. Southeast Asian J Trop Med Public Health.

[90] Melman SD, Steinauer ML, Cunningham C, Kubatko LS, Mwangi IN, Wynn NB (2009). Reduced susceptibility to praziquantel among naturally occurring Kenyan isolates of Schistosoma mansoni. PLoS Negl Trop Dis.

[91] Sadhu PS, Kumar SN, Chandrasekharam M, Pica-Mattoccia L, Cioli D, Rao VJ (2012). Synthesis of new praziquantel analogues: potential candidates for the treatment of schistosomiasis. Bioorg Med Chem Lett.

[92] Patra M, Ingram K, Leonidova A, Pierroz V, Ferrari S, Robertson MN (2013). In vitro metabolic profile and in vivo antischistosomal activity studies of (η(6)-praziquantel)Cr(CO)3 derivatives. J Med Chem.

[93] Portes MC, De Moraes J, Véras LMC, Leite JR, Mafud AC, Mascarenhas YP (2016). Structural and spectroscopic characterization of epiisopiloturine-metal complexes, and anthelmintic activity vs S mansoni. J Coord Chem.

[94] Costa V, Quintanilha A, Moradas-Ferreira P (2007). Protein oxidation, repair mechanisms and proteolysis in Saccharomyces cerevisiae. IUBMB Life.

[95] Xiao SH, Sun J (2017). Schistosoma hemozoin and its possible roles. Int J Parasitol.

[96] Cecarini V, Gee J, Fioretti E, Amici M, Angeletti M, Eleuteri AM (2007). Protein oxidation and cellular homeostasis: emphasis on metabolism. Biochim Biophys Acta.

[97] Ernst A, Stolzing A, Sandig G, Grune T (2004). Antioxidants effectively prevent oxidation-induced protein damage in OLN 93 cells. Arch Biochem Biophys.

[98] Cadet J, Davies KJA (2017). Oxidative DNA damage & repair: an introduction. Free Radic Biol Med.

[99] Halliwell B (2000). Why and how should we measure oxidative DNA damage in nutritional studies?. how far have we come? Am J Clin Nutr.

[100] Gaschler MM, Stockwell BR (2017). Lipid peroxidation in cell death. Biochem Biophys Res Commun.

[101] Mylonas C, Kouretas D (1999). Lipid peroxidation and tissue damage. In Vivo.

[102] Battistelli M, Malatesta M, Meschini S (2016). Oxidative stress to promote cell death or survival. Oxid Med Cell Longev.

